# Isorhamnetin: A Nematocidal Flavonoid from *Prosopis laevigata* Leaves Against *Haemonchus contortus* Eggs and Larvae

**DOI:** 10.3390/biom10050773

**Published:** 2020-05-15

**Authors:** Edgar Jesús Delgado-Núñez, Alejandro Zamilpa, Manasés González-Cortazar, Agustín Olmedo-Juárez, Alexandre Cardoso-Taketa, Ernesto Sánchez-Mendoza, Daniel Tapia-Maruri, David Osvaldo Salinas-Sánchez, Pedro Mendoza-de Gives

**Affiliations:** 1 Centro de Investigación en Biotecnología, Universidad Autónoma de Morelos, Doctorado en Ciencias Naturales (CEIB-UAEM) Av. Universidad 1001 Col. Chamilpa, Cuernavaca, Morelos CP 62209, México; edgarjezus@gmail.com (E.J.D.-N.); ataketa@uaem.mx (A.C.-T.); 2 Unidad Académica, Preparatoria No. 10, Universidad Autónoma de Guerrero, Carretera Nacional Iguala-Taxco, s/n, Iguala, Guerrero CP 40000, México; 3 Centro de Investigación Biomédica del Sur, CIBIS-IMSS, Argentina 1, Col Centro, Xochitepec, Morelos CP 62790, México; azamilpa_2000@yahoo.com.mx (A.Z.); gmanases2000@gmail.com (M.G.-C.); 4 Centro Nacional de Investigación Disciplinaria en Salud Animal e Inocuidad (CENID-SAI-INIFAP), Carretera Federal Cuernavaca-Cuautla No. 8534, Col. Progreso C.P. 62550 Jiutepec, Morelos A.P. 206-CIVAC, México; aolmedoj@gmail.com; 5 Departamento de Sistemas Biológicos, Universidad Autónoma Metropolitana, CDMX CP 04960, México; ersame@yahoo.com; 6 Departamento de Biotecnología, Centro de Desarrollo de Productos Bióticos, Instituto Politécnico Nacional, Carretera Yautepec-Jojutla Km 6, Calle CEPROBI No. 8, Col. San Isidro, Yautepec, Morelos CP 62731, México; dmaruri@ipn.mx; 7 Centro de Investigación en Biodiversidad y Conservación (CIByC-UAEM), Universidad Autónoma del Estado de Morelos, Av. Universidad 1001 Col. Chamilpa, Cuernavaca, Morelos CP 62209, México

**Keywords:** *Prosopis*, flavonoid, isorhamnetin, nematocidal activity, *Haemonchus*

## Abstract

*Haemonchus contortus* affect small ruminants all over the world. Anthelmintics cause resistance, contamination, and a risk of public health. *Prosopis laevigata* is a plant used as a home remedy against many diseases in Mexico. This study arose from a preliminary study where a *P. laevigata* hydroalcoholic extract (*Pl-hae)* showed anthelmintic activity (*aa*) against *H. contortus*. Searching for bioactive compounds (*bac*) with high *aa*, the *Pl-hae* was fractioned obtaining an aqueous (*Aq-F*) and an ethyl acetate fraction (*EtAc-F*), and a flavonoid with *aa* identified as isorhamnetin was obtained from *EtAc-F*. Both fractions were in vitro assessed by the egg hatch test (*eht*) and larval mortality (*lm*) assays. The *bac* obtained from *EtAc-F* were characterised by NMR analysis. The highest *aa* were recorded with *EtAc-F*, resulting in 100% *eht* and 80.45% *lm* at 0.75 and 30 mg/mL, respectively. Alterations in eggs and larvae attributed to isorhamnetin were recorded by environmental scanning electron microscopy, confocal laser scanning and by high-resolution digital-coupled camera. This flavonoid caused 100% *eht* at 0.07 mg/mL after 48 h and 100% *lm* at 7.5 mg/mL after 72 h exposure. Isorhamnetin has promising potential as an anthelmintic against sheep haemonchosis.

## 1. Introduction

*Haemonchus contortus* is the most pathogenic parasitic nematode; it feeds on the blood of animals and causes severe anaemia capable of killing animals [1]. The regular administration of anthelmintic chemical drugs to animals is a common practice used by farmers to control this parasite [2]. However, this strategy has disadvantages; for instance, it promotes anthelmintic resistance in parasites [3]. Furthermore, drug residues can remain in the milk and meat of treated animals [4,5], and once the drugs are eliminated through the urine or faeces of treated animals they can contaminate the soil or water, which is hazardous to the environment [6]. Plants with medicinal properties are increasingly of interest to farmers, who are searching for alternative methods to control the parasites that do not contain the undesirable features possessed by chemical anthelmintic drugs [7]. *Prosopis laevigata* (Fabaceae), also called “Mezquite”, is a leguminous tree found throughout a wide region of arid and semi-arid zones of Mexico and other parts of the world [8]. Medicinal properties, such as antibacterial [9], antimicrobial [10], fungicidal [11], antioxidant [12], and anti-inflammatory [13] activities of the plant were previously recorded. On the other hand, the genus *Prosopis* has been used to treat several diseases including ulcers and parasitosis [14] and it has been also used as a traditional remedy by rural communities of the Paraguaná peninsula in Venezuela to control gastrointestinal parasitic worms [15]. There are only a few studies about the nematocidal activity of *P. laevigata* extracts against ruminant parasitic nematodes. In a study by López-Aroche et al. [16] an important in vitro activity against *H. contortus* infective larvae was reported; and later on, the anthelmintic efficacy of a *P. laevigata n*-hexane extract reduced the *H. contortus* parasitic burden in gerbils that was used as an in vivo model of study [17]. This information opened new expectations to keep ahead with exploring the potential anthelmintic effect of extracts/fractions/compounds of this plant. The objectives of the present study were to evaluate the in vitro nematocidal activity of fractions obtained from a hydroalcoholic extract from *P. laevigata* leaves against eggs and larvae of *H. contortus* as well as and to identify main compounds associated with anthelmintic activity.

## 2. Materials and Methods

### 2.1. Plant Material

The *P. laevigata* leaves (4.6 kg) were collected in the community of Huixastla, Morelos State, Mexico. This area belongs to the Sierra de Huautla Biosphere Reserve, Morelos (18°28′45″ N and 99°8′41″ W). Samples were collected in April and May of 2017. A specimen was deposited at the Herbarium of the Universidad Autónoma del Estado de Morelos (UAEM) (Voucher number: 34873), and it was taxonomically identified by MSc Gabriel Flores Franco, at Centro de Investigación en Biodiversidad y Conservación (CIByC-UAEM). 

### 2.2. Prosopis laevigata Hydroalcoholic Extract Obtaining and Chemical Fractioning

Fresh *P. laevigata* leaves were dried under dark conditions at 25–28 °C for 5d. The dry leaves (4.6 kg) were ground and placed in 46 L crystal containers. Extraction using ground leaves was performed using water-ethanol (70:30%), which was maintained for 24 h at room temperature (18–25 °C). Extracts were filtered through a Whatman no. 4 paper and the solvent was removed by low-pressure distillation using a rotatory evaporator (45–50 °C, Büchi R-300, Flawil, Switzerland) to obtain a semi-solid extract that was finally lyophilised. The extract was kept at 4 °C for biological and phytochemical assays [18]. The hydroalcoholic extract was partitioned using liquid-liquid fractionation (1:1 *v*/*v*) with an immiscible mixture of water and ethyl acetate (1000 mL each, Merck, Germany) giving two fractions: an aqueous (Aq-F) and an ethyl acetate (EtAc-F). Both fractions were dried using low-pressure distillation and were totally dried using the lyophilisation process (Labconco, Kansas, MO, USA). The in vitro anthelmintic activities of the two fractions were evaluated using *H. contortus* eggs and larvae as biological models. 

### 2.3. Isolation, Purification and Identification of Isorhamnetin from the P. laevigata Active Fraction (EtAc-F)

**Scheme 1:**
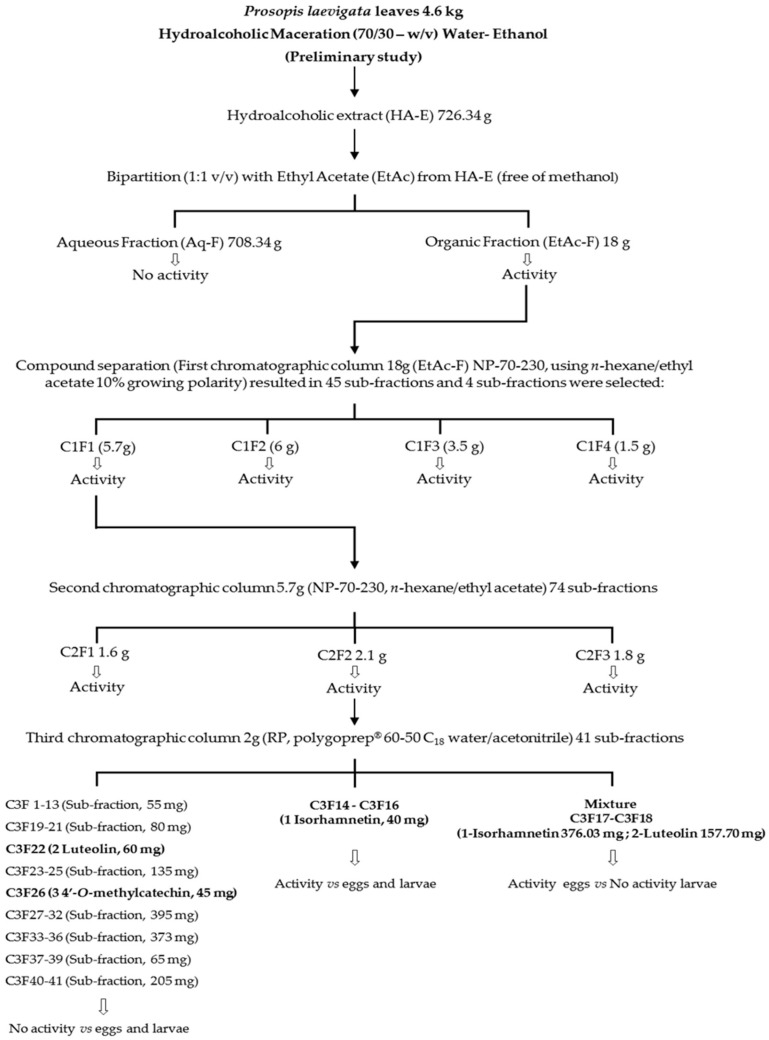
Bio-guided fractioning process to identify compounds with anthelmintic activity from *Prosopis laevigata* leaves against *Haemonchus contortus* eggs and larvae (L_3_).

The most active fraction (EtAc-F, 18 g) was processed using chromatographic techniques with silica gel in an open column (200 g, 70–230 mesh; Merck, Darmstadt, Germany). Then, the solution was eluted using a gradient system with *n*-hexane/ethyl acetate/MeOH, with growing polarity as mobile phase starting with 100% *n*-hexane and ending with 100% methanol. Forty-five, 300 mL fractions were obtained. Fractions were grouped depending on their chemical similarity, which was monitored using thin layer chromatography (TLC) and were concentrated using a rotatory evaporator at low pressure. As a result, four final sub-fractions were obtained and recorded as follows: C1F1 (5.7 g), C1F2 (6.0 g), C1F3 (3.5 g), and C1F4 (1.5 g). All fractions and sub-fractions were analysed via TLC on a silica gel 60 F254 (Merck, Germany) using UV light at 254 and 360 nm. All fractions were assessed via in vitro anthelmintic assays. The most active fraction (C1F1, 5.7 g) was subjected to chromatographic fractionation in a glass column with silica gel. The sample (30 g) was placed on a silica gel column (200 g, 70–230 mesh; Merck, Darmstadt, Germany). A gradient of *n*-hexane and ethyl acetate was used as a mobile phase, from which 74 fractions were obtained. These fractions were grouped according to their similarity as determined by TLC analysis, resulting in three sub-fractions (C2F1, C2F2 and C2F3), which were evaluated in the bioassays. These three sub-fractions resulted with the same high larval mortality activity. After the LC50 analysis, these three sub-fractions resulted without any statistical difference among them; so sub-fraction (C2F2, 2 g) was selected from the other two, because we had a bit more amount of sample of this sub-fraction to keep ahead with its chromatographic process using a normal phase column (30 g, 70–230 mesh; Merck, Germany). A water/acetonitrile gradient system was used as mobile phase, starting with 100% of H_2_O and ending with 100% CH_3_CN to elute the column polygoprep^®^ 60–50 C18, and 41 fractions were collected. Fractions 14, 15 and 16 were combined to eventually yield a solid yellow powder (1, 40 mg). Fractions 17 and 18 produced a mixture of compounds (**1**, isorhamnetin 376.03 mg and **2**, luteolin 157.70 mg giving a total of 533.73 mg), fraction 22 contained compound **2**, (60 mg), meanwhile, the fraction 26 produced compound **3** (45 mg) (See Scheme 1). Compound identification was carried by ^1^H and ^13^C NMR analysis, using an Agilent DD2-600 spectrometer with one NMR probe at 25°C in CD_3_OD and DMSO-D_6_ (Cambridge Isotope Laboratories Inc., Tewksbury, MA, USA) as a solvent and TMS as reference. Note: 1, 2 and 3 NMR spectra are available as Supplementary Materials.

### 2.4. Chemical Group Identification in P. laevigata Hydroalcoholic Extract (HA-E) and Fractions

The *P. laevigata* HA-E and its fractions were phytochemically characterised to determine the presence of alkaloids, volatile coumarins, flavonoids, condensed tannins, terpenes/sterols and saponins (see Chemical group identification as Supplementary Materials). 

### 2.5. Analysis of Extracts, Fractions and Compounds **1**–**3** by HPLC

Chromatographic analysis by HPLC was performed using a Waters 996 HPLC module, equipped with a photodiode array detector and EmpowerPro software (Waters Corporation, Milford, MA, USA). Chemical separation was achieved using a Supelcosil LC-F column (4.6 mm × 50 mm), with a 5-μm particle size, (Sigma-Aldrich, Bellefonte, PA, USA). The mobile phase consisted of 0.5% trifluoroacetic acid aqueous solution (solvent A) and acetonitrile (solvent B) in a gradient system (0–1 min, 0% B; 2–3 min, 5% B; 4–20 min, 30% B; 21–23 min, 50% B; 24–25 min, 80% B; 26–27 100% B; 28–30 min, 0% B). The flow rate was maintained at 0.9 mL/min. The injection volume was 10 μL. The photodiode array detector was set at a wavelength of 310 nm for the identification phenolic compounds.

### 2.6. MS Analysis of Compounds **1**–**3**

Molecular weights of the isolated compounds (**1**–**3**) were determined in Waters triple quadruple mass spectrometer (MS) (Milford MA, EE. UU), equipped with an electrospray ionisation (ESI). The ionisation source was heated to 150 °C. The desolvation temperature was 450 °C and the nitrogen gas flow rate was 900 L/h. Argon was used as a collision gas at a flow rate of 0.10 mL/min (Thermo Fisher Scientific, Bremen, Germany).

### 2.7. Haemonchus contortus Eggs and Larvae Obtaining

The eggs of the parasite were obtained from a *H. contortus* artificially infected lamb (INIFAP strain). One 3-month-old, parasite free, with 22 kg body weight, male lamb, was orally infected with approximately 350 *H. contortus* infective larvae per kg body weight. After a 21-day, pre-patent period, faeces were collected directly from the rectum of the animal. The parasite egg extraction procedure was performed according to von Son-de Fernex et al. [19]. The infective larvae of the parasite were obtained from faecal cultures, using the Baermann technique [20]. After a 7-day incubation period at room temperature (18–25 °C), infective larvae were recovered from faecal cultures using a Baermann funnel apparatus for 12 h. The recovered larvae were washed using differential gradient centrifugation with 40% saccharose solution. Larvae were exsheathed with 0.187% sodium hypochlorite solution. Then, exsheathed larvae were washed by centrifugation with water to eliminate sodium hypochlorite residues. Clean larvae were re-suspended in sterile distilled water and immediately were used for mortality assay. The egg-donor lamb was maintained under controlled conditions according to principles of animal welfare and the elimination of unnecessary animal suffering, which are Good Management Practices policies well established at INIFAP. The Norma Oficial Mexicana (Official Mexican Standard) with official rule number NOM-052-ZOO-1995 (http:/www.senasica.gob.mx), as well as the Ley Federal de Sanidad Animal (Federal Law for Animal Health) DOF 07-06-2012 (https://www.gob.mx/cms/uploads/attachment/file/118761/LFSA.pdf) were strictly abided and all the procedures performed in this study were carried out in accordance with the ethical standards outlined by INIFAP.

### 2.8. Assessment of Larval Mortality by A Bio-Guided Assay

The assay was performed in 96-well microtiter plates (4 wells per treatment). This experiment was performed by triplicate. Treatments were established according to the following sequential steps: Step 1) Aq-F and EtAc-F (10, 20, 30, 40, and 50 mg/mL); Step 2) sub-fractions corresponding to column 1 (C1F1-C1F4) at 3.75–15.00 mg/mL, sub-fractions of column 2 (C2F1-C2F3) at 1.80–15 mg/mL and compounds (**1**, **2** and **3**, as well as a mixture of **1** and **2**) at 0.60–15.00 mg/mL. Each step was assessed using two proper negative controls (distilled water to dissolve Aq-F and 4% methanol to dissolve EtAc-F, sub-fractions, and compounds) and an anthelmintic (ivermectin, 0.5 mg/mL) as a positive control.

Fifty microliters of an aqueous suspension containing approximately 150 *H. contortus* exsheathed infective larvae were deposited into each well (n = 4 wells). Additionally, 50 μL of the corresponding fractions, sub-fractions, compounds, and controls were added to each well. Then, the plates were incubated at room temperature (18–25 °C) for 72 h [21]. After incubation, ten 10-μL aliquots were removed from each well and deposited onto a slide for microscopic examination. Both dead and live larvae were counted according to criteria described by Olmedo-Juárez et al. [18]. The mortality rate was expressed as a mortality percentage and was calculated according to the following formula:

Mortality= [(dead larvae mean)/(live larvae mean + dead larvae mean)] × 100

### 2.9. Assessing the Egg Hatch Test Using P. laevigata Compounds

This assay was carried out in 96-well microtiter plates. Fifty microliters of an aqueous suspension containing approximately 100 *H. contortus* eggs were deposited in each well of the plate (n = 4 wells per treatment). The experiment was performed by triplicate. Fifty microliters of the corresponding compound (**1**, **2** or **3**) were also deposited in their corresponding well. Treatments were established at 0.3, 0.15 and 0.07 mg/mL. Proper controls were used throughout the experiment, which included 2% MeOH and distilled water as negative controls and 0.5 mg/mL of ivermectin as positive control. Plates were incubated at room temperature (18–25 °C) for 48 h [22]. The egg hatch test percentage (EHT) was estimated using a formula described by Coles et al. [23].

*% EHT* = [(number of eggs)/(number of larvae + number of eggs)] × 100

### 2.10. Examination of H. contortus Eggs and Larvae Using Environmental Scanning Electron Microscopy (ESEM) and Confocal Laser Scanning Microscopy (CLSM)

Morphological changes in eggs and larvae post-exposure to bioactive compounds were visualised in detail using two microscopy techniques: ESEM and CLSM. First, ESEM was performed using a Carl Zeiss microscope, Model EVO LS10 (Munich, Germany). Samples were mounted on aluminium stubs and attached using double-sided carbon conductive tape and a retro-disperse electron detector set at 20 kV and 20 Pa pressure. Stubs were directly observed and images were captured at 200× and 1000× magnification in TIFF format (2048 × 1536 pixels). Second, CLSM was performed using A Carl Zeiss microscope, Model LSM 800 (Munich, Germany). Samples were mounted on glass slides and were observed in lambda mode in which a sequence of images was collected at laser wavelengths of 405 nm, 488 nm, 561 nm and 640 nm (4% capacity). The ZEN 2.6 Zeiss Blue edition software was used. Images were taken at 20× and 40× magnification with the Apochromatic Plan, 1.3 numerical opening and 1 Airy Unit (AU) of a pinhole opening. The images were obtained at a 2048 × 2048-pixel resolution in TIFF format. For colour photographs of samples mounted on glass slides and observed via confocal microscopy as previously described were taken using a coupled HD camera (AxioCam, Carl Zeiss, Model 305, colour, Göttingen, Germany)

### 2.11. Statistical Analysis

Egg hatch test and larval mortality percentages were normalised using the square root transformation and analysed based on a completely random design through an analysis of variance (ANOVA). Differences among means were compared using the Tukey test (*p* < 0.05). In addition, 50% and 90% lethal concentrations (LC_50_ and LC_90_) were determined with the PROC PROBIT procedure included within the SAS statistic package [24].

## 3. Results

### 3.1. HA-E and Fraction Yields

Crude hydroalcoholic extract (HA-E) yielded 726.34 g (15.79%) of a brown powder. Additionally, when HA-E was partitioned, EtAc-F and the Aq-F sub-fractions produced yields of 2.48% (18 g) and 97.52% (708.34 g), respectively.

### 3.2. Preliminary Phytochemical Screening of HA-E, Aq-F and EtAc-F

HA-E, Aq-F and EtAc-F fractions from *P. laevigata* leaves contained alkaloids, flavonoids, tannins, triterpenes or sterols, and saponins (Table 1).

### 3.3. Assessment of the Larvicidal Activity of Aq-F and EtAc-F

The nematocidal activity of Aq-F and EtAc-F against *H. contortus* larvae is shown in Table 2. The Aq-F did not show activity against the parasite at the tested concentrations. On the other hand, the EtAc-F produced the highest larvicidal effect and the concentration of 50 mg/mL resulted in 96.01% mortality. 

### 3.4. Larvicidal Activity of Sub-Fractions and Compounds

The mortality percentages corresponding to the sub-fractions C1F1, C1F2, C1F3, C1F4, C2F1, C2F2, C2F3 and compounds **1** isorhamnetin, **2** luteolin, **3** 4´-*O*-methylcatechin and mixture of **1** and **2**); as well as the LC50 are shown in Table 3. The treatments with C1F1, C1F2, C1F3 and C1F4 produced larval mortalities of 79.4%, 24.4%, 6.8% and 5.7%, respectively. On the other hand, the C2F1 and C2F2 sub-fractions resulted in the total larval mortality (100%) at 7.5 mg/mL. Meanwhile, the C2F3 showed mortality close to 95% at the same concentration. Finally, the compound **1** displayed a total larvicidal effect at 7.5 and 15 mg/mL. Likewise, lower concentrations also resulted in high larval mortalities: 3.7 mg/mL = 88.2% and 2.5 mg/mL = 68.15%. Meanwhile, the **2** and **3** as well as the mixture (**1** and **2**) had a null larvicidal effect (Table 3). 

### 3.5. Ovicidal Activity of the Purified Compounds

The luteolin and 4′-*O*-methylcatechin did not show any ovicidal activity. Nevertheless, the mixture of isorhamnetin/luteolin resulted in 100% of ovicidal activity. Meanwhile, the pure compound required solely 0.07 mg/mL to obtain the same total activity (Table 4). This difference indicates that pure isorhamnetin was 5.2 folds more potent than the EtAc-F.

### 3.6. Prosopis laevigata Chemical Analysis Through High Performance Liquid Chromatography (HPLC)

**Figure 1:**
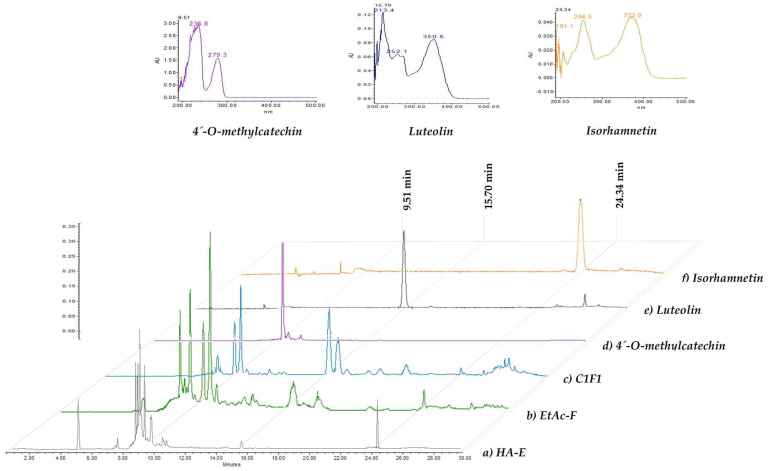
Phytochemical profile visualised by HPLC of (**a**) hydroalcoholic extract (HA-E), (**b**) ethyl acetate fraction (EtAc-F), (**c**) sub-fraction (C1F1), (**d**) 4′-*O*-methylcatechin, (**e**) luteolin and (**f**) isorhamnetin.

The HPLC chromatograms from compounds responsible for the nematocidal activity of *P. laevigata* HA-E and EtAc-F are shown in Figure 1. The HA-E contained at least six compounds, including phenolic compounds with retention times of 4.5 and 8.3 min and four flavonoids (8.6, 8.9, 9.5 and 25.6 min). The EtAc-F chromatogram was used to identify flavonoid-type compounds (8.7, 9.0, 9.5, 10.0, 10.3 and 15.7 min) (Figure 1b). The C1F1 sub-fraction chromatogram as well as the identified compounds (isorhamnetin, luteolin and 4′-*O*-methylcatechin) and isorhamnetin of the EtAc-F chromatographic fraction are shown in Figure 1c–f.

### 3.7. Chemical Structures of Identified Compounds

**Figure 2:**
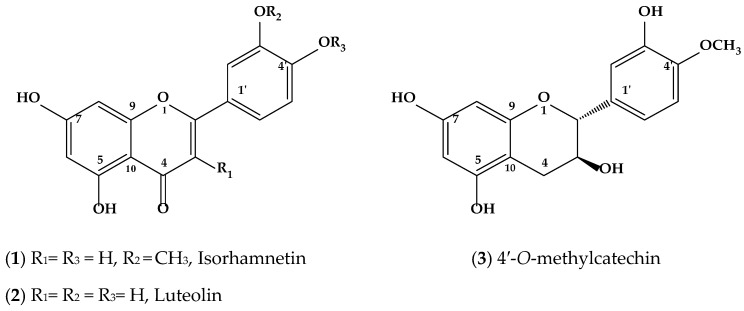
Chemical compound structures (**1**, **2** and **3**) obtained from *p. laevigata* leaves.

The analysis of spectra allowed researchers to identify compounds present in sub-fractions of *P. laevigata* extracts and revealed the presence of three flavonoids, isorhamnetin, an isorhamnetin-luteolin mixture, and luteolin. Furthermore, 4′-*O*-methylcatechin, which is a polyphenolic compound, was identified (Figure 2).

Compound (**1**) was isolated as a yellow powder. Its UV spectrum revealed that the compound had a λmax at 254 and 373 nm, which is a characteristic of a flavonoid. Negative ion EI-MS of compound **1**, produced quasimolar ion peaks at m/z 316 [M + H]^-^ (calcd. for C_16_H_12_O_7_, 316.05) (See isorhamnetin mass spectra, as Supplementary Material). The ^1^H NMR spectra of **1** showed two aromatic systems: an AB system (δ 6.19 (1H, d, 1.9 Hz, H-6) and 6.47 (1H, d, 2.4 Hz, H-8)] and ABX system [δ 7.75 (1H, d, 2.3 Hz, H-2′), 6.94 (1H, d, 8.4 Hz, H-5′) and 7.68 (1H, dd, 2.4, 8.4 Hz, H-6′)). In addition, a signal corresponding to an oxygenated base proton was observed in δ 3.8 that integrates three protons and associates HSQC with the carbon signal in δ 55.7, which is characteristic of methoxyl groups at the C-3′ position. On the basis of these data, and direct comparisons with spectroscopic data described in the literature, compound (**1**) was identified as isorhamnetin [25].

Once isolated, compound (**2**) was an orange powder with a UV λmax at 205, 255 and 368 nm, which is a distinguishing characteristic of flavonols. The negative ion EI-MS of (**2**) revealed a quasimolar ion peak at m/z of 286 [M-H]^-^ (calcd. for C_15_H_10_O_6_, 286.05) (see luteolin mass spectra, in the Supplementary Material). Analysis of the ^1^H and ^13^C NMR spectra of compound (**2**) showed that that compound was similar to (**1**), since it displayed the same chemical shift signals, except that compound (**2**) lacked a signal corresponding to the methoxyl group at C-3′, indicating that its identity was luteolin (**2**) [26].

Isolation of compound (**3**) resulted in the production of a pink powder. This compound displayed a UV absorption spectrum between λmax values of 236 and 279 nm; a characteristic of catechin. In the negative ion EI-MS of **3**, a quasimolar ion peak was calculated at m/z 304 [M-H]^-^ (calcd. for C_16_H_16_O_6_, 304.09) (See 4′-*O*-methylcatechin mass spectra, as Supplementary Material). The analysis of the ^1^H and ^13^C NMR spectra showed characteristic signals of 4′-*O*-methylcatechin (**3**) [27]. 

**Isorhamnetin** (**1**): ^1^H NMR (600 MHz, DMSO-*d_6_*); δ 6.19 (1H, d, 1.9 Hz, H-6), 6.47 (1H, d, 2.4 Hz, H-8), 7.75 (1H, d, 2.3 Hz, H-2′), 6.94 (1H, d, 8.4 Hz, H-5′), 7.68 (1H, dd, 2.4, 8.4 Hz, H-6′), 3.8(OCH_3_), 12.4(1H, s, 5-OH); ^13^C NMR (150 MHz, DMSO-d6); δ 146.6 (C-2), 135.8 (C-3), 175.8 (C-4), 160.6 (C-5), 98.2 (C-6), 163.9 (C-7), 93.5 (C-8), 156.1 (C-9), 103.0 (C-10), 121.9 (C-1′), 111.7 (C-2′), 147.3 (C-3′), 148.7 (C-4′), 115.5 (C-5′), 121.7 (C-6′), 55.7 (3′-OCH_3_).

**Luteolin** (**2**): ^1^H NMR (600 MHz, DMSO- *d_6_*); δ 6.19 (1H, d, 2.0 Hz, H-6), 6.44 (1H, d, 2.0 Hz, H-8), 6.67 (1H, s, H-3), 7.39 (1H, d, 2.0 Hz, H-2′), 6.89 (1H, d, 8.6 Hz, H-5′), 7.41 (1H, dd, 2.0, 8.1 Hz, H-6′), 12.9(1H, s, 5-OH); ^13^C NMR (150 MHz, DMSO-d6); δ 146.6 (C-2), 102.8 (C-3), 181.6 (C-4), 161.4 (C-5), 98.8 (C-6), 164.1 (C-7), 93.8 (C-8), 157.2 (C-9), 103.7 (C-10), 121.5 (C-1′), 113.3 (C-2′), 145.7 (C-3′), 149.6 (C-4′), 116.0 (C-5′), 118.9 (C-6′).

**4′-*O*-Methylcatechin** (**3**): ^1^H-NMR (600 MHz, DMSO- *d_6_*); δ 4.51 (1H, d, 7.5 Hz, H-2), 3.82 (1H, m, H-3), 2.65 (1H, dd, 5.1, 15.9 Hz, H-4a), 2.35 (1H, dd, 8.3, 15.9 Hz, H-4b), 5.69 (1H, d, 2.3 Hz, H-6), 5.89 (1H, d, 2.3 Hz, H-8), 6.76 (1H, d, 1.9 Hz, H-2′), 6.87 (1H, d, 8.3 Hz, H-5′), 6.72 (1H, dd, 1.9, 8.3 Hz, H-6′), 3.75 (OCH_3_); ^13^C-NMR (150 MHz, DMSO-d6); δ 80.7 (C-2), 66.3 (C-3), 27.8 (C-4), 156.1 (C-5), 93.8 (C-6), 156.4 (C-7), 95.14 (C-8), 155.2 (C-9), 99.0 (C-10), 132.2 (C-1′), 114.3 (C-2′), 146.1 (C-3′), 147.1 (C-4′), 111.8 (C-5′), 118.2 (C-6′), 55.6 (4′-OCH_3_). 

### 3.8. Examination of H. contortus Eggs and Larvae ESEM and CLSM

**Figure 3:**
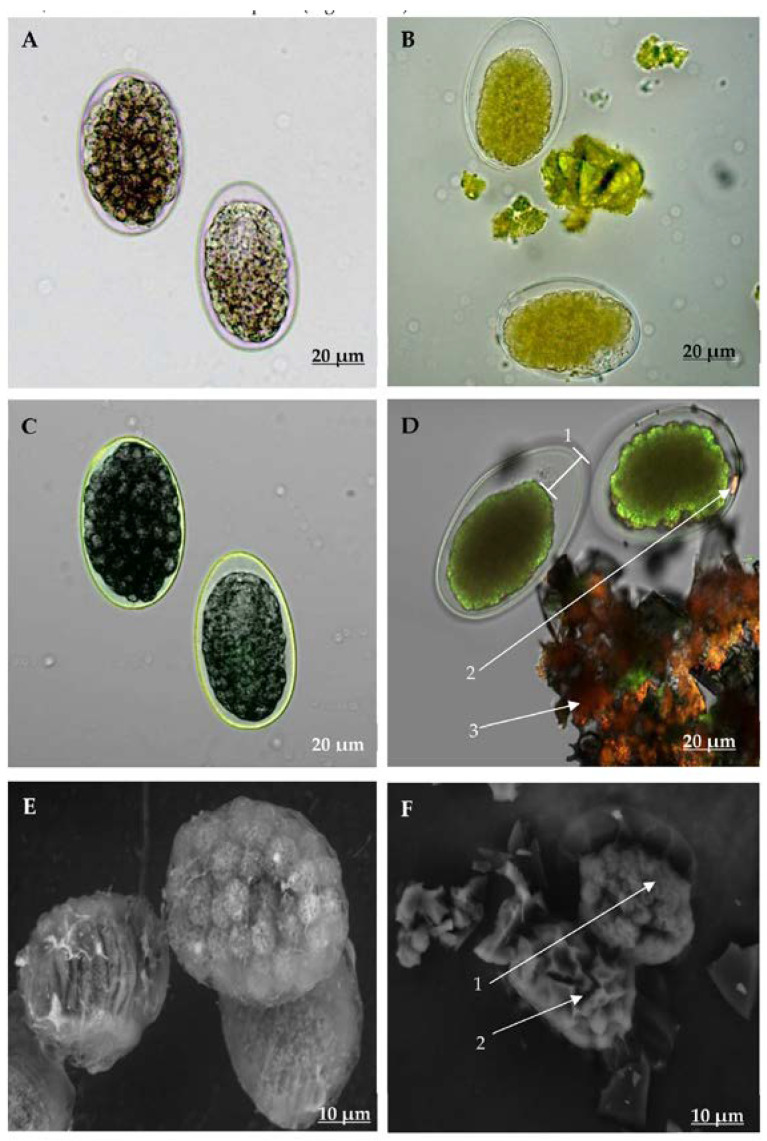
Micrographs taken with AxioCam (**A**,**B**), *Confocal Laser Scanning Microscopy* (CLSM, **C**,**D**) and *Environmental Scanning Electron Microscopy* (ESEM, **E**,**F**), showing the aspect of *Haemonchus contortus* eggs exposed (**B**,**D**,**F**) and non-exposed to isorhamnetin (**A**,**C**,**E**).

General characteristics and structural differences between non-exposed and isorhamnetin-exposed eggs (at 7.5 mg/mL) are shown in Figure 3. AxioCam and CLSM images revealed that eggs that had not been exposed to isorhamnetin had normal internal and external structures, and their morula cells occupied almost the entire inner cavity of the eggshell as expected (Figure 3A,C). On the other hand, the eggs exposed to isorhamnetin displayed significant structural changes, which included the interruption of their embryonic development, and a cell mass reduction of the egg and eggshell of about 30% (Figure 3D-1). Additionally, some eggs had irregular edges and deformations that produced an appearance of wrinkled surface (Figure 3B,D). 

CLSM micrographs of isorhamnetin-exposed eggs revealed autofluorescence that was emitted in the morula at wavelengths of 515 and 560 nm (Figure 3). This autofluorescence was not identified in the eggshell, which remained clear. This feature allowed us to identify a superposition between the fluorescence emitted by morula cells and fluorescence emitted by isorhamnetin using chromophore fragments that were fixed to the morula cell surface (Figure 3D-2). It is important to note that CLSM allowed us to visualise isorhamnetin fluorescence, which appeared in orange colour and was emitted between 560 nm and 600 nm (Figure 3D-3). ESEM further revealed that eggs that were not exposed to isorhamnetin had a smooth eggshell surface, and the morula developed normally (Figure 3E). In contrast, the isorhamnetin-exposed eggs exhibited a loss of cell architectural structure (Figure 3F-1). Deformations observed in these eggs included the presence of cracks on the morula, as well as intracellular spaces (Figure 3F-2).

**Figure 4:**
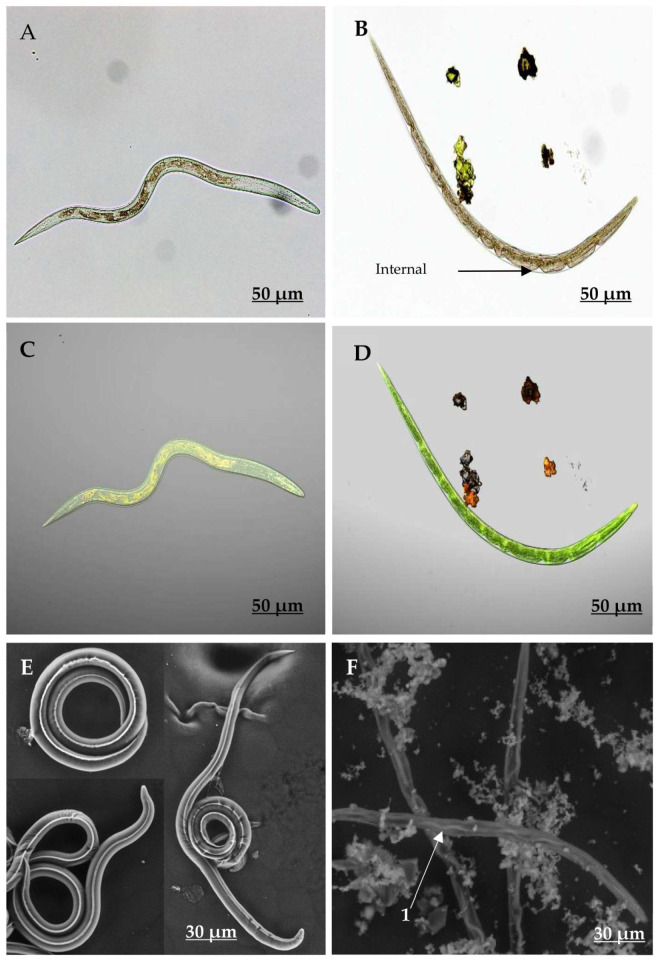
Micrographs taken with AxioCam (**A**,**B**), *Confocal Laser Scanning Microscopy* (CLSM, **C**,**D**) and *Environmental Scanning Electron Microscopy* (ESEM, **E**,**F**) showing the aspect of *Haemonchus contortus* larvae exposed (**B**,**D**,**F**) and non-exposed to isorhamnetin (**A**,**C**,**E**). F1 shows the wave formations at the lateral nervous cord.

Differences observed using AxioCam CLSM and ESEM between non-exposed and isorhamnetin-exposed larvae (at 7.5 mg/mL) are shown in Figure 4. Non-exposed larvae displayed a sharpness and depth that resulted from the capacity of light to freely pass through their bodies. Furthermore, no apparent changes in their body conformation, intestinal cell organisation or in their external cuticle, were observed (Figure 4A,C,E). In contrast, the bodies of isorhamnetin-exposed larvae showed some changes in their internal organs, including the loss of architectural integrity of intestinal cells (Figure 4B). Likewise, some morpho-anatomic changes were observed in the isorhamnetin-exposed larvae ie., the presence of an irregular dark colour that diffused throughout the larval body length as well as some irregular pale spaces between the surface coat and the inner coat that resembled tissue folds (Figure 4B,D). The ESEM of the isorhamnetin non-exposed larvae showed a normal smooth cuticular surface without any apparent abnormal changes (Figure 4E). In contrast, the bodies of isorhamnetin-exposed larvae appeared deformed, with loss of internal organ integrity (Figure 4F). In addition, these larvae also showed changes in their external cuticle, which appeared rough and straight with wavy formations mainly in their lateral nervous cords along the body (4 F-1). Furthermore, the transversal size of exposed larvae was reduced in comparison with non-exposed larvae (Figure 4F). 

## 4. Discussion

The results of the present study revealed that *P. laevigata* leaves possess compounds that are active against *H. contortus* infective larvae. It is interesting the fact that the pure isorhamnetin showed 100% larval mortality when used at 15 and 7.5 mg/mL and this effect decreased when concentration decreased and even at 2.5 mg/mL this sole compound provoked 68.15% LM. In contrast the other two identified compounds luteolin and 4′-*O*-methylcatechin both at 2.5 mg/mL resulted in 0% activity. Since our point of view; although isorhamnetin is not the major compound the results of the present study clearly indicate that isorhamnetin is definitively the compound responsible for the anthelmintic effect.

On the other hand, when the mixture isorhamnetin + luteolin at same concentration (2.5 mg/mL) was assessed, a null effect was also recorded, which suggests that luteolin act as an antagonist of isorhamnetin, which inhibited the isorhamnetin larval mortality effect.

Likewise, it is important to remark that the ovicidal activity showed by the pure isorhamnetin at different low concentrations was also very high. It is interesting to emphasise the fact that luteolin showed two different effects when combined with isorhamnetin; since the luteolin inhibited the larval mortality of the isorhamnetin; however, it did not influence the high ovicidal activity of isorhamnetin. A possible explanation about this fact could be that perhaps the differences in the membrane receptors between the eggshell and the cuticle coat could interfere either at favour or against the activity of this compound; although this is only a proposal that will have to be studied in depth. The fact that the isorhamnetin caused 100% EHT at such as low concentrations is a very good characteristic that makes it a potential candidate for future in vivo studies. It is interesting the fact that both C2F2 sub-fraction and isorhamnetin at 15 and 7.5 mg/mL respectively, caused 100% larval mortality. However, we actually do not have a categorical explanation why the C2F2 that is a mixture of different compounds, including 20% isorhamnetin showed the same high percentage of larval mortality when compared with sole isorhamnetin. Other studies should be designed to clarify this question. 

Previous assays performed in our laboratory showed an important nematocidal effect of the hydroalcoholic extract from this arboreal legume (unpublished data). In an extend review of the literature we found little information regarding the nematocidal activity of *P. laevigata* extracts against *H. contortus* which was solely related to activity against the larvae of the parasite. Therefore, as far as we know, the present study is the first report of the ovicidal activity of and organic ethyl acetate fraction extracts from *P. laevigata.*

Several metabolites from other *Prosopis* species were identified using different parts of the plant and with different solvent systems. The nematocidal activity of these groups of metabolites has been previously described in other *Prosopis* species and plant extracts from other genera. It is worth mentioning that plants from the *Prosopis* genus, and other genera and species of plants, displayed nematocidal activity against different nematodes of importance for agriculture and livestock industry. In this context, is clear that independently of the genus/specie fabaceae plants, they respond with a different biological activity pattern, regardless the separation system used. 

Likewise, it is important to consider that exploring other separation strategies would be beneficial to improve the bioactive compounds obtained [28]; for example, Fourier Transform Infrared Spectroscopy (FTIR) has demonstrated significant value in characterizing and identifying bioactive compounds [29].

A methanolic extract obtained from fruit from other Fabaceae plant, *Caesalpinia coriaria,* resulted in 73% larval mortality at 150 mg/mL [30]. In our study, the *P. laevigata* EtAc-F resulted in 96% in vitro larval mortality against *H. contortus* at only 50 mg/mL after 72 h exposure. These mortalities were similar to other results obtained with a *Lysiloma acapulcensis* EtAc-F, which belongs to the same taxonomic group that caused 100% mortality against *H. contortus* infective larvae at the same concentration and same exposure time [31]. These authors reported the same larvicidal inefficacy of *L. acapulcensis* Aq-F that we found with *P. laevigata* Aq-F. The phytochemical screening showed different groups of compounds, including flavonoids, tannins, alkaloids, and coumarins. It is interesting that the compound with the highest nematocidal activity was a phenolic compound (flavonoid). 

The HPLC analysis revealed that HA-E contained phenolic compounds (Figure 1a), while EtAc-F contained hydroxycinnamic acid derivate compounds and flavonoids (Figure 1b). It is important to mention that isorhamnetin is a flavonoid compound that has been previously isolated from several plant species including *Persicaria glabra* [25] and *Cleome africana* [32]. 

Isorhamnetin possesses several biological activities, including cytotoxic [33], anti-microbial [34], antiviral [35], anti-oxidant [36,37], hepato-protective and cardio-vascular protective [38,39], neurological [40], anti-cancer [41], anti-inflammatory [42,43] and anti-diabetes [44]. However, this seems to be the first report of the anthelmintic activity of isorhamnetin against parasitic nematodes affecting small ruminants.

Use of AxioCam, CLSM and ESEM techniques supported the hypothesis that isorhamnetin is the compound responsible for producing the morphological structural changes associated with pathogenic effects against eggs and larvae of the parasite. The use of AxioCam, CLSM and ESEM allowed identifying the presence of interesting changes in the morphological structure of eggs and larvae associated with the presence of the isorhamnetin. The mechanism of the anthelmintic activity of isorhamnetin against *H. contortus* eggs and larvae remains uncertain and will be motive of future studies. 

The flavonoids generally interact with lipidic bilayers in membranes and may affect biological/physiological processes i.e.., protein transportation. Beyond this, they may act as substrates, which interfere with the electric properties of membranes [45,46]. Some authors reported that phenolic compounds, including condensed tannins and flavonoids are capable of binding to membrane proteins of the eggshell that are vital for optimal development and biological activities of larvae [47]. However, membranes of nematodes such as *H. contortus* are constituted of three different types of layers that include an external protein layer, a medial layer comprised of chitin fibrils and proteins (for eggs and larvae, respectively), and a semi-permeable internal lipid layer [48,49]. The egg hatching processes are initiated as a result of an environmental stimulus that promotes a protein/enzyme-mediated process that triggers the release of larva from the eggshell. The proteins that promote egg hatching are called “hatching enzymes” and they include proteases, lipases, beta-glucosidases, chitinases and leucine aminopeptidases, which directly act to promote eggshell membrane degradation [48,50]. It is believed that the compound-protein interactions may cause important structural changes that affect membrane permeability, oxygen interchange, and the release of substances and enzymes that promote eggshell degradation to eventually promote larval release [47,51,52]. Isorhamnetin, which is a flavonoid compound, could act similarly via binding to membrane receptors of the parasite in order to cause damage in nematode tissues; however, this is only a hypothesis that must be validated using more advanced techniques.

Molan et al. [53] and Lakshmi et al. [54] reported that phenolic compounds including condensed tannins and flavonoids possess important anthelmintic activities. For instance, Barrau et al. [55] noted that tri-glycosylated flavonols isolated from *Onobrychis viciifolia* Scop. inhibited the larval migration in *H. contortus* under in vitro conditions. Later on, Klongsiriwet et al. [56] demonstrated the synergic effects of a combination of condensed tannins and flavonoids, which included quercetin and luteolin. This combination improved the anthelmintic activity. One of the main hypotheses regarding the mechanism of action of these compounds states that they may promote eggshell and larval cuticle permeability [52,57]. Nevertheless, the mode of entry of the phenolic compounds into the membrane depends on their chemical structure. This occurs through the formation of hydrogen bonds among the groups of lipids containing polar head groups and hydrophilic portions of flavonoids as well as with hydroxyl groups that interact with parasitic membranes [58,59,60]. Larvae exposed to isorhamnetin displayed altered surface coat characteristics. The fact that the external surfaces of eggshells were not significantly affected, suggests that entry of isorhamnetin alone or combined with luteolin into the eggshell membrane occurs without producing these effects despite the capacity of isorhamnetin to cause embryo death. Finally, because of our assays used eggs and infective larvae of *H. contortus* as targets of the *P. laevigata* compounds and that both are free-living stages of the parasite, it is important to design complementary experiments about the impact of isorhamnetin on non-target nematodes of the soil.

## 5. Conclusions

The ethyl acetate fraction obtained from a hydroalcoholic extract of *P. laevigata* leaves possess a potent anthelmintic activity against *H. contortus* eggs and infective larvae, which is considered the most economically important parasite affecting small ruminants globally. Chromatographic procedures facilitated the isolation and identification of three flavonoid compounds (**1**–**3**). Of these, isorhamnetin possessed the highest degree of anthelminthic activity. CLSM and ESEM techniques revealed important structural features of parasites exposed to isorhamnetin. This is the first report of the in vitro anthelmintic activity of isorhamnetin against *H. contortus.*

## Figures and Tables

**Table 1:** Groups of metabolites identified from *Prosopis laevigata* leaves using general chemical reaction assays.

Metabolite Group	Reagent	Colorimetric Reaction	AH-E	Aq-F	EtAc-F
**Alkaloids**	Dragendorff		+++	+++	+
	Mayer	Turbidity or precipitate	+++	+++	+
	Wagner	(red to orange, white to cream and brown)	+++	+++	+
**Coumarins**	Bornträger	Yellow fluorescence (UV)	-	-	-
**Flavonoids**	Mg^2+^ and HCL	Red, orange and violet	++	-	+++
**Tannins**	Ferric chloride	Hydrolysable tannins (blue)	-	-	-
		Condensed tannins (green)	+++	+	+++
	**Confirmation**				
	Solution of gelatine	Precipitate white	+	+	+
	Gelatine and saline solution	Precipitate white	+	+	+
	Saline solution	Precipitate white	-	-	-
**Triterpenes/sterols**	Liebermann-Burchard Salkowski Salkowski (modified)	Green, blue-red (sterols) Yellow, red to purple (triterpenes) Yellow to red (triterpenoidal/steroidal saponins)	- - +	- - +	+ + +
**Saponins**	Water	Foam formation	+	++	-

(-) Not detected (+) light positive reaction (++) positive reaction (+++) strong positive reaction.

**Table 2:** Results of the in vitro lethal assessment of two fractions from *Prosopis laevigata* leaves, aqueous fraction (Aq-F) and ethyl acetate fraction (EtAc-F) against *Haemonchus contortus* infective larvae (L_3_) after 72 h confrontation.

Treatment	Mean of Recovered Larvae Dead Larvae/Total	% Mortality (±SD)
First step (Fractioning for larvicidal activity)		
Aqueous fraction (Aq-F) (mg/mL)		
50	0.75/105.62	0.72 ± 0.67 ^g^
40	0.62/104.4	0.60 ± 49 ^g^
30	0/104.25	0 ^g^
20	0/104.62	0 ^g^
10	0/103.87	0 ^g^
Ethyl acetate fraction (EtAc-F) (mg/mL)		
50	72.5/75.3	96.01 ± 0.75 ^b^
40	93.5/101.25	92.32 ± 0.87 ^c^
30	81.8/101.55	80.45 ± 0.83 ^d^
20	82.5/109.87	75.13 ± 0.81 ^e^
10	34.3/110.55	31.12 ± 0.76 ^f^
Distilled water	0.7/86.7	0.9 ^g^
MeOH (4%)	1.5/69.4	2.98 ± 0.26 ^f^
Ivermectin (0.5 mg/mL)	81/81	100 ^a^
Variation coefficient		1.17
R^2^		0.99

Note: Means with different letter into same column indicate statistic differences (*p* < 0.05); MeOH = Methanol; SD = Standard Deviation.

**Table 3:** In vitro mortality percentages and lethal concentration (95%) attributed to the effect of four *Prosopis laevigata* sub-fractions and compounds derived from EtAc-F against *Haemonchus contortus* infective larvae (L_3_).

Sub-Fractions and Compound (Step 2)	Mean of Recovered Larvae Dead Larvae/Total	% Mortality ±SD	Lethal Concentrations 95% (CI Limits Lower-Upper)	
C_1_F1 (mg/mL)			LC_50_ (mg/mL)	LC_90_ (mg/mL)
15	67.5/84.75	79.47 ^b^		
7.5	18.15/76.75	24.12 ^d^		
3.7	8/77.2	10.47 ^d^		
C_1_F2 (mg/mL)				
15	11.2/70	24.45 ± 2.44 ^d^		
C_1_F3 (mg/mL)				
15	4.5/66.25	6.82 ± 0.72 ^e^		
C_1_F4 (mg/mL)				
15	3/78.5	5.75 ± 2.56 ^e^		
C_2_F1 (mg/mL)				
15	127.5/0	100 ^a^	2.82 (2.72–2.92)	3.82 (3.69–3.96)
7.5	126.5/0	100 ^a^		
3.75	107.5/122.5	87.8 ± 3.46 ^b^		
1.87	2/120	2.27 ± 0.45 ^e^		
C_2_F2 (mg/mL)				
15	120.0/0	100 ^a^	2.68 (2.45–2.86)	3.84 (3.67–4.02)
7.5	121/0	100 ^a^		
3.7	101.5/115	88.2 ± 0.47 ^b^		
1.8	10.75/107.5	10.0 ± 0.77 ^d^		
C_2_F3 (mg/mL)				
15	117/0	100 ^a^	3.04 (2.89–3.19)	5.66 (5.32–6.07)
7.5	109.7/116.5	94.22 ± 1.63 ^b^		
3.7	77.7/115.2	67.47 ± 1.29 ^c^		
1.8	2.5/115.2	2.15 ± 0.51 ^e^		
Isorhamentin (mg/mL) (**1**)				
15	126/0	100 ^a^	2.07 (1.98–2.16)	3.79 (3.60–4.02)
7.5	127/0	100 ^a^		
3.7	101.5/115	88.25 ± 0.47 ^b^		
2.5	73.25/107.5	68.15 ± 4.46 ^c^		
1.2	14.5/106.75	13.6 ± 3.19 ^d^		
0.6	2.25/105.75	2.15 ± 0.50 ^e^		
Isorhanmetin and luteolin (2.5 mg/mL) (**1**,**2**)	0/103.82	0 ^f^		
Luteolin (2.5 mg/mL) (**2**)	0.5/107.2	0.47 ± 0.95 ^f^		
4′-*O*-methylcatechin (2.5 mg/mL) (**3**)	0/104.2	0 ^f^		
Distilled water	0/71.25	0 ^f^		
MeOH (4%)	0/69.87	0 ^f^		
Ivermectin (0.5 mg/mL)	73.5/73.5	100 ^a^		
Variation coefficient		3.56		
R^2^		0.99		

Note: Means inside same column with different letter indicate significant differences (Tukey test) *p* < 0.05; MeOH = Methanol, SD = Standard deviation; CI = Confidence Interval.

**Table 4:** Results of EHT and lethal concentrations (LC_50_ and LC_90_) of compounds obtained from *Prosopis laevigata* leaves at different concentrations against *Haemonchus contortus* eggs after 48 h interaction.

Treatments	%EHT ± SD	Lethal Concentrations 95% (CI limits Lower-Upper)	
		LC_50_ (mg/mL)	LC_90_ (mg/mL)
Isorhamnetin (mg/mL) (**1**)			
0.3	100 ^a^		
0.15	100 ^a^		
0.07	100 ^a^		
			
Mixture isorhamnetin and luteolin (mg/mL) (**1**,**2**)			
0.3	100 ^a^		
0.15	96.03 ± 1.36 ^b^	0.10	0.13
0.07	4.18 ± 1.23 ^c^	(0.09–0.10)	(0.13–0.14)
			
Luteolin (mg/mL) (**2**)			
0.3	0 ^c^		
0.15	0 ^c^		
0.07	0 ^c^		
	
4′-*O*-methylcatechin (mg/mL) (**3**)			
0.3	0 ^c^		
0.15	0 ^c^		
0.07	0 ^c^		

MeOH 2%	1.50 ± 0.6 ^c^		
Ivermectin (0.5 mg/mL)	100 ^a^		
Variation coefficient	2.92		
R^2^	0.99		

Note: Means into same column with different literals indicate significant differences *p* < 0.05; CI = Confidence Interval; SD = Standard deviation; MeOH = Methanol.

## Supplementary Materials

The following are available online at https://www.mdpi.com/2218-273X/10/5/773/s1, Supplementary data: Chemical group identification.
