# Correction: Delgado-Núñez et al. Isorhamnetin: A Nematocidal Flavonoid from *Prosopis laevigata* Leaves Against *Haemonchus contortus* Eggs and Larvae. *Biomolecules* 2020, *10*, 773

**DOI:** 10.3390/biom16030357

**Published:** 2026-02-27

**Authors:** Edgar Jesús Delgado-Núñez, Alejandro Zamilpa, Manasés González-Cortazar, Agustín Olmedo-Juárez, Alexandre Cardoso-Taketa, Ernesto Sánchez-Mendoza, Daniel Tapia-Maruri, David Osvaldo Salinas-Sánchez, Pedro Mendoza-de Gives

**Affiliations:** 1 Centro de Investigación en Biotecnología, Universidad Autónoma de Morelos, Doctorado en Ciencias Naturales (CEIB-UAEM) Av. Universidad 1001 Col. Chamilpa, Cuernavaca, Morelos CP 62209, México; edgarjezus@gmail.com (E.J.D.-N.); ataketa@uaem.mx (A.C.-T.); 2 Unidad Académica, Preparatoria No. 10, Universidad Autónoma de Guerrero, Carretera Nacional Iguala-Taxco, s/n, Iguala, Guerrero CP 40000, México; 3 Centro de Investigación Biomédica del Sur, CIBIS-IMSS, Argentina 1, Col Centro, Xochitepec, Morelos CP 62790, México; azamilpa_2000@yahoo.com.mx (A.Z.); gmanases2000@gmail.com (M.G.-C.); 4 Centro Nacional de Investigación Disciplinaria en Salud Animal e Inocuidad (CENID-SAI-INIFAP), Carretera Federal Cuernavaca-Cuautla No. 8534, Col. Progreso C.P. 62550 Jiutepec, Morelos A.P. 206-CIVAC, México; aolmedoj@gmail.com; 5 Departamento de Sistemas Biológicos, Universidad Autónoma Metropolitana, CDMX CP 04960, México; ersame@yahoo.com; 6 Departamento de Biotecnología, Centro de Desarrollo de Productos Bióticos, Instituto Politécnico Nacional, Carretera Yautepec-Jojutla Km 6, Calle CEPROBI No. 8, Col. San Isidro, Yautepec, Morelos CP 62731, México; dmaruri@ipn.mx; 7 Centro de Investigación en Biodiversidad y Conservación (CIByC-UAEM), Universidad Autónoma del Estado de Morelos, Av. Universidad 1001 Col. Chamilpa, Cuernavaca, Morelos CP 62209, México

There was an error in the original publication [1]. References [28] and [29] were incorrectly used. These references pertain to the separation of lignin from cellulose using hydrogen peroxide techniques to enhance biofuel production, which is unrelated to the content of the paragraph in question. Therefore, the references are now replaced by the two new ones:

28. Abedelmaksoud, T.G.; Younis, M.I.; Altemimi, A.B.; Tlay, R.H.; Ali Hassan, N. Bioactive Compounds of Plant-Based Food: Extraction, Isolation, Identification, Characteristics, and Emerging Applications. *Food Sci. Nutr.* **2025**, *13*, e70351. https://doi.org/10.1002/fsn3.70351. PMID: 40501497; PMCID: PMC12152400.
29. Sasidharan, S.; Chen, Y.; Saravanan, D.; Sundram, K.M.; Yoga Latha, L. Extraction, isolation and characterization of bioactive compounds from plants’ extracts. *Afr. J. Tradit. Complement. Altern. Med.* **2011**, *8*, 1–10. PMID: 22238476; PMCID: PMC3218439.

The paragraph was accordingly revised and added below.

“Likewise, it is important to consider that exploring other separation strategies would be beneficial to improve the bioactive compounds obtained [28]; for example, Fourier Transform Infrared Spectroscopy (FTIR) has demonstrated significant value in characterizing and identifying bioactive compounds [29].”

The authors state that the scientific conclusions are unaffected. This correction was approved by the Academic Editor. The original publication has also been updated.
